# Pharmacological Prevention of Postoperative Delirium: A Systematic Review and Meta-Analysis of Randomized Controlled Trials

**DOI:** 10.1155/2019/9607129

**Published:** 2019-03-14

**Authors:** Yong Liu, Xiao-Jin Li, Yi Liang, Yan Kang

**Affiliations:** ^1^Department of Intensive Care Unit, West China Hospital of Sichuan University, Chengdu, China; ^2^Department of Anesthesiology, Affiliated Hospital of Guilin Medical University, Guilin, China

## Abstract

**Background:**

The high prevalence of delirium among postoperative patients has increased morbidity and mortality. The kind of drug that can effectively reduce the incidence of delirium has become the focus of discussion in recent years. However, a consensus in this respect has yet to be reached.

**Methods:**

Randomized controlled trials (RCTs) were retrieved from the PubMed, Cochrane Library, ClinicalTrials.gov, and Embase databases from their inception through October 12, 2018. We included RCTs of pharmacological prevention for postoperative delirium in adults (at least 18 years), and the Cochrane risk of bias tool was used to evaluate the methodological quality of trials. The primary outcomes were the risk ratios (RRs) of incidence of postoperative delirium, and the secondary outcomes were the RRs of mortality and adverse events in the intervention and control groups.

**Results:**

Thirty-eight trials, which comprised 20302 patients and 18 different drugs, were included in the analysis. Of the 38 studies, 17 were rated as low risk with respect to methodological quality. Dexmedetomidine administration (RR 0.58, 95%CI 0.44-0.76, P<0.01) was associated with a significantly lower incidence of postoperative delirium than the control conditions. However, the findings from the studies with a low risk of bias did not show a significant difference in this beneficial effect (RR 0.64, 95%CI 0.39-1.04, P=0.07). The antipsychotic drugs olanzapine (RR 0.44, 95%CI 0.30- 0.65, P<0.01) and risperidone (RR 0.42, 95%CI 0.19-0.92, P=0.03) had promising effects, but there was a lack of sufficient evidence to obtain a definitive conclusion. The beneficial effect of other drugs, including haloperidol, methylprednisolone, dexamethasone, gabapentin, ketamine, cyproheptadine, donepezil, hypertonic saline, melatonin, nimodipine, ondansetron, pregabalin, rivastigmine, TJ-54, and tryptophan, was not proven on the basis of present evidence.

**Conclusion:**

Among the pharmacological prophylactic measures for postoperative delirium, dexmedetomidine, olanzapine, and risperidone showed higher efficacy than other drugs. However, more high-quality evidence is needed to confirm these results.

## 1. Introduction

Delirium, a change in neuropsychiatric state from a previous baseline level of mental function, typically involves a set of symptoms such as changes in arousal, cognitive deficits, and perceptual dysfunction, as well as hallucinations and delusions. Delirium itself is not a disease but rather a set of symptoms. Delirium not only is a challenge for medical staff but also has adverse effects on the duration of the hospital stay and mechanical ventilation and the cognitive state, and delirium contributes to increased morbidity and mortality.

Several classes of drugs, such as *α*_2_-receptor agonists, atypical antipsychotics, and sleep-regulatory drugs, have received widespread attention for the potential prevention or treatment of delirium [1].

Dexmedetomidine, an agonist of *α*2-adrenergic receptors in certain parts of the brain, is an anxiolytic, sedative, and modest analgesic [2, 3]. Dexmedetomidine has been promoted for its ability to achieve sedation without risk of respiratory depression (unlike other commonly used sedatives such as midazolam and propofol) and can achieve levels of semiarousable and cooperative sedation. However, the administration of dexmedetomidine has been associated with hypertension and arrhythmia due to peripheral *α*2-receptor stimulation [4].

Atypical antipsychotics are less likely to cause extrapyramidal side effects, such as body rigidity, bradykinesia, and involuntary tremors, than haloperidol, one of the most widely used typical antipsychotics [5–7]. Although atypical antipsychotics are deemed safer than typical antipsychotics, they still have the potential to induce severe side effects in accordance with their respective side effect profiles, and they more commonly increase the risk of metabolic side effects, such as weight gain and glycemic and lipid imbalances.

Melatonin, a hormone secreted by the pineal gland, is regarded as an important molecular sleep–wake cycle regulator that is used to treat insomnia [8]. Some studies have shown that low or delayed melatonin levels in elderly patients are associated with delirium in intensive care units [9–11]. Several RCTs have been registered and are ongoing to prove the benefits of melatonin in preventing postoperative delirium.

A number of randomized controlled trials (RCTs) have been published focusing on the pharmacological prevention of postoperative delirium. This systematic review was performed to identify recent advances in the pharmaceutical prophylaxis of postoperative delirium and to offer clinicians an updated summary to help make clinical decisions.

## 2. Materials and Methods

### 2.1. Retrieval Protocol and Selection Criteria

We searched MEDLINE, Web of Science, the Cochrane Central Register of Controlled Trials, ClinicalTrials.gov, and Embase through October 12, 2018, for RCTs investigating the prevention of postoperative delirium. We also examined the reference lists of the included relevant RCTs and systematic reviews for additional eligible references. Search terms mainly included delirium, confusion, disorientation, surgery, and RCTs.

RCTs that investigated the pharmacological prevention of postoperative delirium were included, with language restricted to English. Patients were adults (at least 18 years of age) and received drugs in the perioperative phase. Studies were excluded if risk ratios (RRs) for analysis were not available or if they investigated the therapeutic effects of the drugs for emergency agitation and anesthesia. The studies in which several drugs were simultaneously used to prevent postoperative delirium were also excluded.

### 2.2. Data Extraction and Quality Assessment

Data extraction was conducted independently by the 1st and 2nd authors (Liu Y and Liang Y) with a predesigned spreadsheet, and discrepancies were resolved by a 3rd author (Li XJ).

The Cochrane risk of bias tool was used to assess the risk of bias, with items including random sequence generation, allocation concealment, blinding of participants and personnel, blinding of outcome assessment, and incomplete outcome data [12]. A risk of bias table was created to display the results of the risk assessment.

### 2.3. Primary and Secondary Results

The primary outcomes were the RRs of the incidence of postoperative delirium between the intervention and control groups after the patients received the drugs, and the secondary outcomes were the RRs of mortality and adverse events. Other results, such as adverse events, side effects, and hospital stays, were also collected for evaluating the safety of the drugs. To maintain consistency between studies with regard to the control groups, only studies using placebo, normal saline, and blank (meaning “no injection”) as control agents were included in the final data analysis.

### 2.4. Statistical Analysis

All statistical analyses were completed by Stata 13.0 (Stata Corp., College Station, TX). Considering the clinical heterogeneity between studies, the random effects model using the DerSimonian and Laird method was used to merge data. The heterogeneity was evaluated using the I^2^ statistic, and I^2^ > 30% indicated the presence of heterogeneity between studies [13]. Subgroup analyses were adopted to identify the effect of different characteristics of the studies on the results. Publication bias was assessed by Egger's asymmetry test and funnel plots [14]. This systematic review and meta-analysis was conducted in accordance with the Preferred Reporting Items for Systematic Reviews and Meta-Analyses (PRISMA) checklist [15].

## 3. Results

### 3.1. Search Results and Study Characteristics

**Figure 1:**
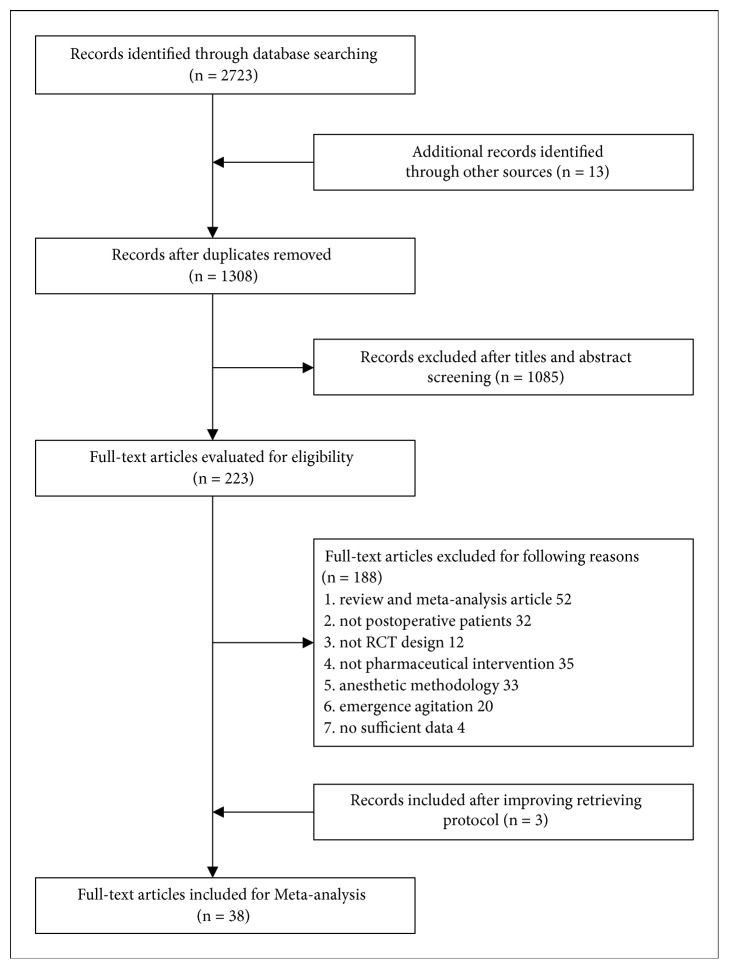
Flow diagram of the study selection process.

We identified 2723 records, of which 1308 were duplicates (Figure 1). Of the 223 full-text articles reviewed, 38 RCTs were identified as eligible after improving the retrieval protocol [16–53]. Baseline information is listed in Table 1. To maintain consistency between studies, 32 studies involving 19539 patients (including 34 datasets) treated with placebo, normal saline, or blank as controls were included in the final data analysis.

### 3.2. Quality Assessments

**Figure 2:**
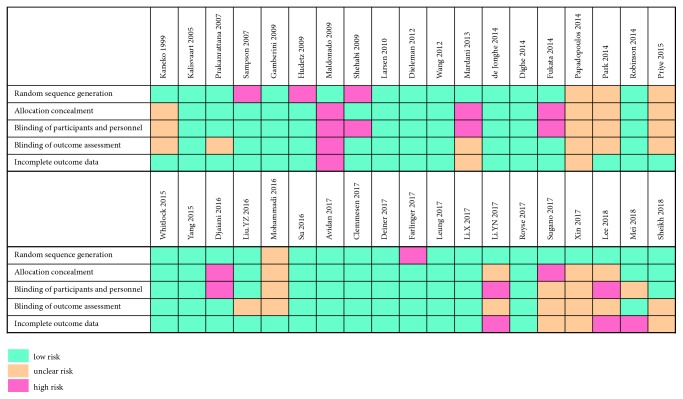
Summary of risk of bias assessment.

The overall methodological quality of the studies was distributed from low to high (Figure 2). Five items, including random sequence generation, allocation concealment, blinding of participants and personnel, blinding of outcome assessment, and incomplete outcome data, were adequately and unambiguously described in 31 (82%), 25 (66%), 23 (61%), 24 (63%), and 29 (76%) of 38 trials, respectively.

### 3.3. Prophylactic Efficacy Assessments

**Figure 3:**
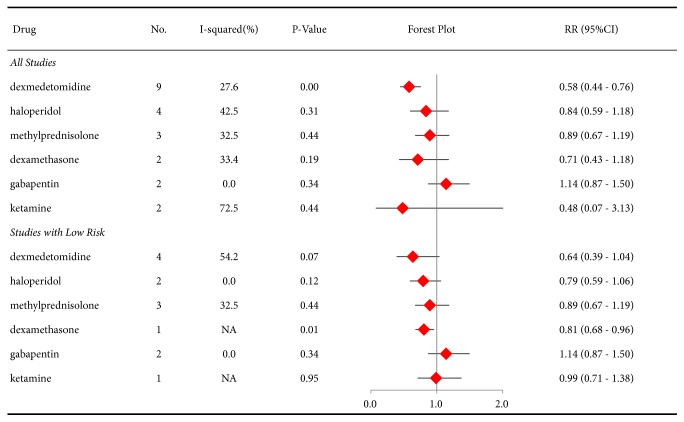
Forest plot of risk ratios (RRs) for the incidence of postoperative delirium in all studies or studies with a low risk of bias (at least 2 studies for each drug).

Out of concern about the risk of bias, the RRs for the incidence of postoperative delirium were analyzed at two levels: studies with different levels of bias risk and studies with low risk. First, drugs that were investigated in at least two studies were evaluated, and we found that dexmedetomidine (RR 0.58, 95%CI 0.44-0.76, P<0.01) was associated with the beneficial effect of decreasing the incidence of postoperative delirium, but haloperidol, methylprednisolone, dexamethasone, gabapentin, and ketamine did not display this effect (Figure 3). In contrast, the results of only the studies with low risk showed that dexamethasone (RR 0.81, 95%CI 0.68-0.96, P=0.01) showed a beneficial benefit, while the effects of dexmedetomidine (RR 0.64, 95%CI 0.39-1.04, P=0.07), haloperidol, methylprednisolone, gabapentin, and ketamine were not significantly different from those of controls.

**Figure 4:**
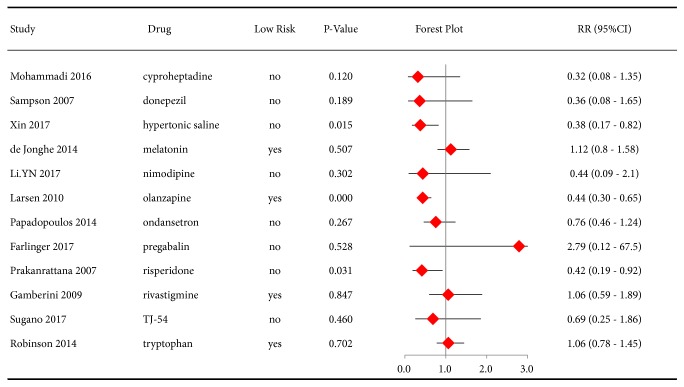
Forest plot of risk ratios (RRs) for the incidence of postoperative delirium (only one available study for each drug).

The preventive effect of drugs with 1 eligible study on postoperative delirium was also evaluated. Olanzapine (RR 0.44, 95%CI 0.30-0.65, P<0.01) and risperidone (RR 0.42, 95%CI 0.19-0.92, P=0.03) had protective effects in the prevention of delirium, but cyproheptadine, donepezil, hypertonic saline, melatonin, ondansetron, rivastigmine, TJ-54, and tryptophan did not (Figure 4).

**Figure 5:**
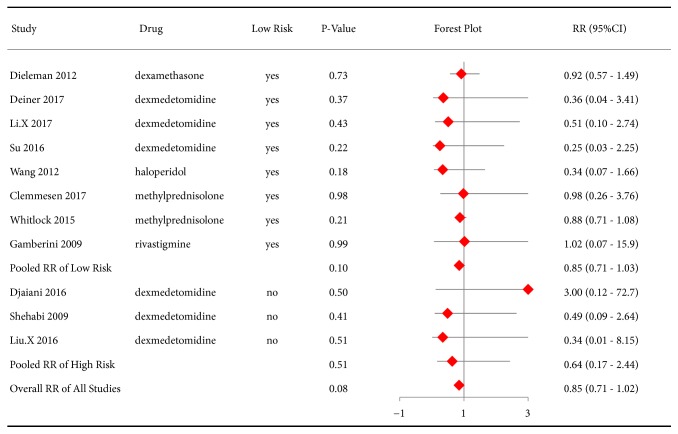
Forest plot of risk ratios (RRs) for mortality in the included studies.

The prophylactic effect of drugs on overall mortality was assessed in our review. The RR from all studies did not show a significant difference between the intervention and control groups (RR 0.85, 95%CI 0.71-1.02, P=0.08). Merging data from the 8 studies with a low risk had a similar result (RR 0.85, 95%CI 0.71-1.03, P=0.10) (Figure 5).

Adverse events and side effects were also collected to evaluate the balance between the benefits and risks produced by these drugs. We found that dexmedetomidine increased the incidence of bradycardia (RR 1.24, 95%CI 1.01-1.52, P=0.04) and reduced the incidence of tachycardia (RR 0.51, 95%CI 0.32-0.82, P=0.01) and hypertension (RR 0.67, 95%CI 0.52-0.87, P<0.01). Significant differences in adverse events and side effects were not found with the atypical antipsychotics, the acetylcholinesterase inhibitors, ketamine, and the glucocorticoids, as well as for other effects of dexmedetomidine, in part because of insufficient data (Table S2, supplementary materials).

### 3.4. Subgroup Analysis

We performed the subgroup analysis against only dexmedetomidine because there were 9 datasets, and the other drugs did not have enough data for further analyses. When the datasets were categorized by type of surgery, age, methodological quality, and timing of drug administration, we found that dexmedetomidine had clear protective effects in patients from datasets without cardiac surgery, aged > 65 years, and with insufficient quality. The timing of drug administration, before surgery or after surgery, did not influence postoperative delirium (Table S1, supplementary materials).

### 3.5. Publication Bias

Egger's test for asymmetry, an indication of publication bias, was performed for all the studies, and P=0.001 indicated significant publication bias among the included studies (Fig.  S1, supplementary materials). Nevertheless, the publication bias among studies with low risk did not show a significant difference with P=0.30 (Fig.  S2, supplementary materials).

## 4. Discussion

In this review, we retrieved 38 RCTs from 2723 records investigating the pharmaceutical prevention of postoperative delirium, and 32 of these studies used placebo, saline, or blank as a control. We also systematically evaluated these RCTs of drugs to prevent delirium after surgery, and the overall results showed that *α*2-adrenergic receptor agonists and atypical antipsychotics could reduce the incidence of postoperative delirium. However, there were no drugs that showed an ability to prevent postoperative delirium based on the evidence from studies with low risk.

Although dexmedetomidine had the advantage of reducing postoperative delirium, the results obtained when all studies, regardless of quality level, were examined were inconsistent with the results obtained when only the high-quality studies were examined. This difference means that a definitive conclusion could not be drawn due to the lack of high-quality evidence. It should be noted that dexmedetomidine has both sedative and analgesic effects, which means that the use of dexmedetomidine can reduce the consumption of other sedative drugs and opioid analgesics, which possibly changes the incidence of delirium in patients and limits our ability to interpret the results [54, 55]. In view of the high risk of delirium with benzodiazepines, dexmedetomidine is deemed to be an alternative to benzodiazepines to achieve the target sedation [56]. High-quality evidence is still needed to determine whether dexmedetomidine can truly reduce the occurrence of delirium when compared with placebo.

Atypical antipsychotics have the risk of serious side effects, such as acute hemorrhagic pancreatitis, status epilepticus, leucopenia, tardive dyskinesia, and neuroleptic malignant syndrome [57]. Although the incidence of these severe adverse events with atypical antipsychotics is lower than that with typical antipsychotics, there is not enough evidence to put atypical antipsychotics into widespread use to prevent delirium in all susceptible patients because of the potential adverse effects [58]. Therefore, the pros and cons of using antipsychotics to prevent and treat delirium need be balanced, and the therapeutic regimens must also be tailored according to the specific situation of individual patients.

This review also has some limitations. First, the relevant information provided by the authors and the evaluation process featured subjectivity, which might lead to a certain degree of deviation from the real situation. Second, some studies had not documented in detail the adverse events and side effects caused by the drugs, which may result in difficulties in weighing the risks and benefits of drug use. Third, although the average age of the patients in 2 of the studies in this review was less than 60 years old, we did not think this difference would influence the results, considering the consistency of the baseline between the intervention and control groups.

Dexmedetomidine and two atypical antipsychotic drugs (olanzapine and risperidone) showed prophylactic effects on postoperative delirium. However, the results of the meta-analysis of all studies on dexmedetomidine were inconsistent with the results from the low-risk studies, and there was not enough evidence to support the use of atypical antipsychotics for preventing delirium. Therefore, we need to carefully understand these results and develop reasonable regimens for delirium prevention according to the specific situation.

## Figures and Tables

**Table 1:** Summary of the baseline characteristics of included trials.

Study	Country	Dosing Information	Control	Begin Time	Following Time	Type of Surgery	Age (years)	Male (%)	Incidence (%)	N
Avidan 2017 [16]	USA	0.5 or 1.0 mg/kg ketamine by injection as single dose.	placebo	before surgery	3d	major surgery	70	62.2	19.6	672

Clemmesen 2017 [17]	Denmark	125 mg methylprednisolone by injection for once.	saline	before surgery	3d	hip	80	35.9	24.8	117

de Jonghe 2014 [18]	Netherlands	3 mg melatonin taken orally for 5 days.	placebo	before surgery	8d	hip	84	29.9	27.5	378

Deiner 2017 [19]	USA	0.5 *μ*g/kg/h dexme by infusion continued until 2 hours into recovery.	saline	before surgery	30d	cardiac	74	48.7	15.4	390

Dieleman 2012 [20]	Netherlands	1 mg/kg dexamethasone by injection for once.	placebo	during surgery	30d	cardiac	66	72.5	10.4	4482

Dighe 2014 [21]	Canada	200 mg gabapentin taken orally tid for 4 days.	placebo	after surgery	NA	knee	63	49.7	10.6	161

Djaiani 2016 [22]	Canada	a bolus of 0.4 *μ*g/kg dexme followed by 0.2-0.7 *μ*g/kg/h infusion for maximum 24 h.	propofol	after surgery	5d	cardiac	73	75.4	24.6	183

Farlinger 2018 [23]	Canada	150 mg pregabalin preoperatively and 75 mg bid postoperatively for 7 days.	placebo	before surgery	NA	hip	60	50.9	0.6	163

Fukata 2014 [24]	Japan	2.5 mg haloperidol for 3 days.	blank	after surgery	7d	abdominal, orthopedic	80	52.9	37.2	121

Gamberini 2009 [25]	Switzerland	1.5 mg oral rivastigmine tid for 7 days.	placebo	before surgery	6d	cardiac	74	68.1	31	113

Hudetz 2009 [26]	USA	0.5 mg/kg ketamine intravenous bolus for once.	saline	before surgery	5d	cardiac	64	NA	17.2	58

Kalisvaart 2005 [27]	Netherlands	0.5 mg haloperidol tid until 3 days after surgery	saline	before surgery	14d	hip	79	20.2	15.8	430

Kaneko 1999 [28]	Japan	5 mg haloperidol intravenous for 5 days	saline	after surgery	5d	gastrointestinal	73	64.1	21.8	78

Larsen 2010 [29]	USA	5 mg of orally-disintegrating olanzapine or placebo just before and after surgery.	placebo	before surgery	8d	joint	74	45.8	27.5	400

Lee 2018 [30]	Korea	1 *μ*g/kg bolus dexme followed by 0.2-0.7 *μ*g/kg/h infusion during surgery.	saline	before surgery	5d	laparoscopic	73	44.3	17.9	318

Leung 2017 [31]	USA	900 mg gabapentin administered preoperatively and for the first 3 postoperative days.	placebo	before surgery	3d	orthopedic	73	49.6	22.4	697

Li.X 2017 [32]	China	0.6 *μ*g/kg dexme for 10 minutes followed by 0.4 *μ*g/kg/h during surgery.	placebo	before surgery	5d	cardiac	67	68.6	6.3	287

Li.YN 2017 [33]	China	7.5 *μ*g/kg/h nimodipine was injected continually 30minutes before anesthesia induction.	saline	before surgery	7d	spine	70	40	11.7	60

Liu. YZ 2016 [34]	China	0.2-0.4 *μ*g/kg/h dexme during surgery	saline	before surgery	7d	joint	73	48.7	29.4	197

Maldonado 2009 [35]	USA	received one of three postoperative sedation regimens: dexme, propofol and midazolam.	propofol, midazolam	after surgery	3d	cardiac	58	63.8	34.4	90

Mardani 2013 [36]	Iran	8 mg of intravenous dexamethasone before surgery and followed by 8 mg q8h for 3 days.	placebo	before surgery	3d	cardiac	62	NA	18.3	93

Mei 2018 [37]	China	a bolus of dexme at 0.8-1.0 *μ*g/kg followed by 0.1-0.5 *μ*g/kg/h infusion or propofol.	propofol	before surgery	7d	hip	75	45.6	11.8	296

Mohammadi 2016 [38]	Iran	4 mg cyproheptadine tid for 7 days.	placebo	after surgery	7d	non-cardiac	60	65	25	40

Papadopoulos 2014 [39]	Greece	8 mg intravenous ondansetron daily for 5 days.	placebo	after surgery	5d	femoral, hip	72	44.3	44.3	106

Park 2014 [40]	Korea	loading dose of 0.5 *μ*g/kg dexme followed by 0.2-0.8 *μ*g/kg/h or remifentanil 1-2.5 mg/h during ICU.	remifentanil	after surgery	3d	cardiac	53	55.6	16.2	142

Prakanrattana 2007 [41]	Thailand	1 mg risperidone sublingually when regained consciousness	placebo	after surgery	3d	cardiac	61	58.7	21.4	126

Priye 2015 [42]	India	0.4 *μ*g/kg/h dexme for 12 hours.	saline	after surgery	ICU	cardiac	43	51.6	9.4	64

Robinson 2014 [43]	USA	1g tryptophan enterally tid for 3 days.	placebo	after surgery	ICU	mixed	69	98	38.5	301

Royse 2017 [44]	Australia	250 mg methylprednisolone at induction and 250 mg methylprednisolone before surgery.	placebo	during surgery	3d	cardiac	74	64.1	9.2	298

Sampson 2007 [45]	UK	5 mg donepezil after surgery and continuing for a further 3 days.	placebo	after surgery	4d	hip	69	51.5	21.2	33

Shehabi 2009 [46]	Australia	received dexme 0.1–0.7g/kg/h or morphine 10-70g/kg/h to maintain target sedation & analgesia.	morphine	after surgery	5d	cardiac	71	75.3	11.7	299

Sheikh 2018 [47]	India	1 *μ*g/kg dexme bolus followed by infusion 0.2–0.6 *μ*g/kg/h or 0.25–1 mg/kg/h propofol	propofol	before surgery	ICU	cardiac	35	NA	13.3	60

Su 2016 [48]	China	dexme 0.1 *μ*g/kg/h from ICU admission on the day of surgery until 08:00 h on postoperative day 1.	saline	after surgery	7d	non-cardiac	74	60.4	15.9	700

Sugano 2017 [49]	Japan	2.5g TJ-54 tid for 7 days prior to surgery and 4 days after surgery, except for the operation day.	blank	before surgery	NA	gastrointestinal, lung	77	64.5	8.1	186

Wang 2012 [50]	China	0.5 mg haloperidol intravenous bolus followed by infusion at a rate of 0.1 mg/h for 12 hours.	saline	after surgery	7d	non-cardiac	74	41.1	19.3	457

Whitlock 2015 [51]	Canada	250 mg methylprednisolone at anaesthetic induction and 250 mg at initiation of cardiopulmonary bypass.	placebo	during surgery	3d	cardiac	67	60.4	7.8	7507

Xin 2017 [52]	China	4ml/kg 7.5% hypertonic saline was given before surgery.	saline	before surgery	3d	hip	76	51.7	25	120

Yang 2015 [53]	China	0.5 mg/kg dexme was given for 1 hour.	saline	before surgery	5d	free flap	50	53.2	8.9	79

Note: dexme: dexmedetomidine; N: the number of participants; NA: not available.

## Data Availability

All original data used to support the findings of this study are available from the corresponding author upon request.

## Supplementary Materials

Supplementary Materials Table S1: subgroup analyses of studies using dexmedetomidine to prevent postoperative delirium. Table S2: the incidence of adverse events in the intervention and control groups. Figure S1: the funnel plots of the included studies regardless of the risk of bias. Figure S2: the funnel plots of the included studies with low risk.
