# Supplementary material for: Pharmacological Prevention of Postoperative Delirium: A Systematic Review and Meta-Analysis of Randomized Controlled Trials
Source: Evid Based Complement Alternat Med. 2019 Mar 14;2019:9607129. doi: 10.1155/2019/9607129 (PMC6437723; doi:10.1155/2019/9607129)

Supplementary Materials

Table S1: Subgroup analyses of studies using dexmedetomidine to prevent postoperative delirium.

Subgroup	RR (95%CI)	No. of Datasets	I-squared (%)	P-Value	Weight (%)
Type of Surgery					
non-cardiac	0.58 (0.43 - 0.79)	7	41.0	0.000	91.3
cardiac	0.55 (0.24 - 1.28)	2	0.0	0.168	8.7
Age					
elderly	0.59 (0.44 - 0.79)	7	40.5	0.000	95.7
non-elderly	0.34 (0.10 - 1.22)	2	0.0	0.098	4.3
Quality of Studies					
low risk	0.64 (0.39 - 1.04)	4	54.2	0.072	51.3
high risk	0.54 (0.39 - 0.76)	5	0.0	0.000	48.7
Timing of Administration					
before surgery	0.64 (0.48 - 0.86)	7	21.1	0.003	75.6
after surgery	0.44 (0.30 - 0.65)	2	0.0	0.000	24.4
Overall	0.58 (0.44 - 0.76)	9	27.6	0.000	100

Table S2: The incidence of adverse events in intervention group and control group.

Types of Drugs	Adverse Events	RR (95%CI)	P	No.	Studies
Atypical Antipsychotics	atrial fibrillation	2.05 (0.52 - 8.08)	0.31	1	Larsen 2010 [29]
	arrhythmia	1.13 (0.42 - 3.03)	0.81	2	Larsen 2010 [29], Prakanrattana 2007 [41]
	renal failure	0.68 (0.12 - 3.92)	0.66	1	Prakanrattana 2007 [41]
Acetylcholinesterase Inhibitors	diarrhoea	1.15 (0.48 - 2.76)	0.76	2	Sampson 2007 [45], Gamberini 2009 [25]
	dizziness	2.61 (0.32 - 21.2)	0.37	1	Sampson 2007 [45]
	Insomnia	1.07 (0.69 - 1.67)	0.75	2	Sampson 2007 [45], Gamberini 2009 [25]
	nausea/vomiting	1.12 (0.81 - 1.57)	0.49	2	Sampson 2007 [45], Gamberini 2009 [25]
	parastheseae	0.75 (0.05 - 11.1)	0.83	1	Sampson 2007 [45]
	pyrexia	0.75 (0.05 - 11.1)	0.83	1	Sampson 2007 [45]
	atrial fibrillation	0.9 (0.56 - 1.45)	0.67	1	Gamberini 2009 [25]
	arrhythmia	1.02 (0.21 - 4.84)	0.98	1	Gamberini 2009 [25]
	stroke	0.52 (0.05 - 5.55)	0.59	1	Gamberini 2009 [25]
Dexmedetomidine	arrhythmia	0.89 (0.72 - 1.10)	0.27	2	Li.X 2017 [32], Su 2016 [48]
	bradycardia	1.24 (1.01 - 1.52)	0.04	4	Li.X 2017 [32], Su 2016 [48], Yang 2015 [53], Deiner 2017 [19]
	hypertension	0.67 (0.52 - 0.87)	0.00	2	Su 2016 [48], Deiner 2017 [19]
	hypotension	1.10 (0.94 - 1.29)	0.23	4	Li.X 2017 [32], Su 2016 [48], Yang 2015 [53], Deiner 2017 [19]
	infection	3.00 (0.13 - 71.5)	0.50	1	Yang 2015 [53]
	nausea/vomiting	0.87 (0.29 - 2.65)	0.81	1	Yang 2015 [53]
	stroke	1.02 (0.26 - 4.03)	0.98	2	Li.X 2017 [32], Deiner 2017 [19]
	tachycardia	0.51 (0.32 - 0.82)	0.01	1	Su 2016 [48]
Ketamine	atrial fibrillation	0.94 (0.55 - 1.6)	0.82	1	Avidan 2017 [16]
	hallucination	1.26 (0.90 - 1.75)	0.18	1	Avidan 2017 [16]
	hypotension	0.9 (0.44 - 1.85)	0.78	1	Avidan 2017 [16]

	nausea/vomiting	0.95 (0.77 - 1.17)	0.62	1	Avidan 2017 [16]
	tachycardia	0.99 (0.43 - 2.27)	0.98	1	Avidan 2017 [16]
Glucocorticoids	atrial fibrillation	0.96 (0.91 - 1.02)	0.23	3	Dieleman 2012 [20], Mardani 2013 [36], Whitlock 2015 [51]
	mycocardial infarction	0.93 (0.59 - 1.45)	0.74	2	Dieleman 2012 [20], Mardani 2013 [36]
	pneumonia	1.07 (0.15 - 7.53)	0.95	2	Dieleman 2012 [20], Mardani 2013 [36]
	renal failure	0.84 (0.69 - 1.03)	0.09	2	Dieleman 2012 [20], Whitlock 2015 [36]
	resepiratory failure	0.86 (0.71 - 1.03)	0.10	3	Dieleman 2012 [20], Mardani 2013 [36], Whitlock 2015 [51]
	stroke	0.89 (0.69 - 1.17)	0.40	3	Dieleman 2012 [20], Mardani 2013 [36], Whitlock 2015 [51]
	gastrointestinal heamorrhage	1.19 (0.81 - 1.76)	0.38	1	Whitlock 2015 [51]
	infection	0.95 (0.84 - 1.07)	0.39	1	Whitlock 2015 [51]

Figure S1: The funnel plots of the included studies regardless of the risk of bias.

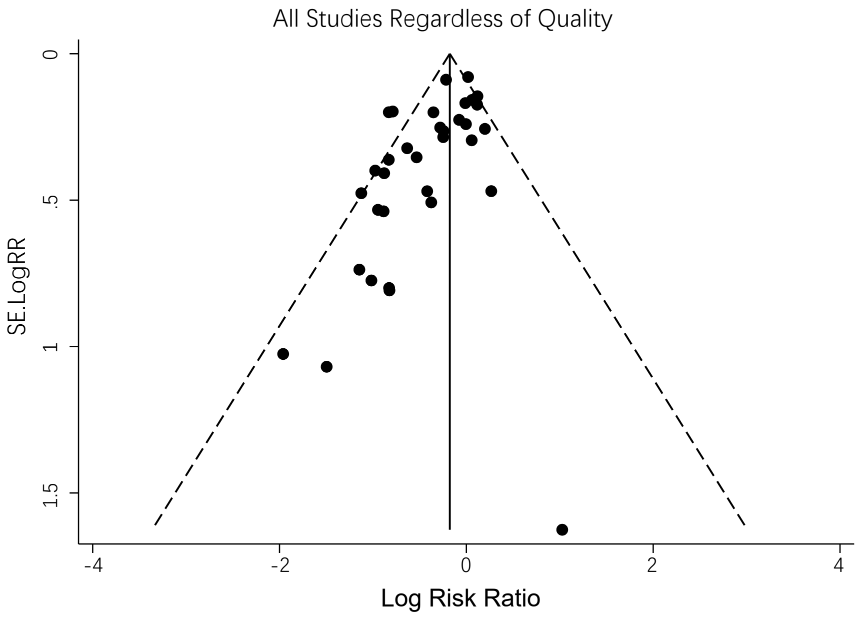

Figure S2: The funnel plots of the included studies with low risk.

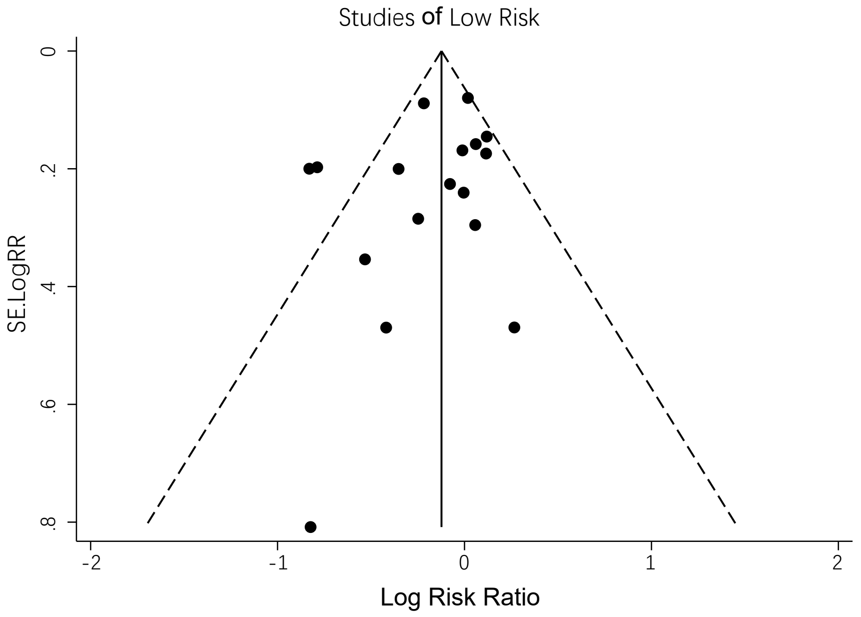

Supplement: Supplementary Materials — Table S1: subgroup analyses of studies using dexmedetomidine to prevent postoperative delirium. Table S2: the incidence of adverse events in the intervention and control groups. Figure S1: the funnel plots of the included studies regardless of the risk of bias. Figure S2: the funnel plots of the included studies with low risk. [file 9607129.f1.pdf]
